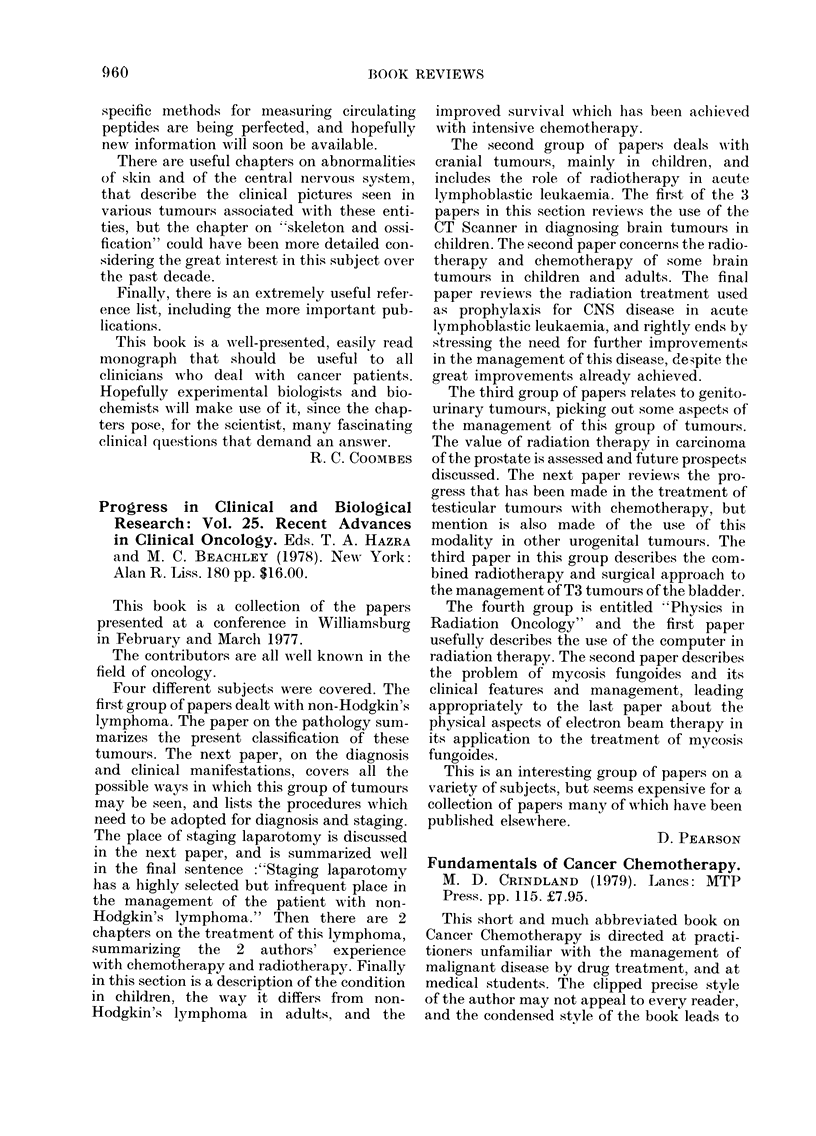# Progress in Clinical and Biological Research: Vol. 25. Recent Advances in Clinical Oncology

**Published:** 1979-12

**Authors:** D. Pearson


**Progress in Clinical and Biological Research: Vol. 25. Recent Advances in Clinical Oncology.** Eds. T. A. Hazra and M. C. Beachley (1978). New York: Alan R. Liss. 180 pp. $16.00.

This book is a collection of the papers presented at a conference in Williamsburg in February and March 1977.

The contributors are all well known in the field of oncology.

Four different subjects were covered. The first group of papers dealt with non-Hodgkin's lymphoma. The paper on the pathology summarizes the present classification of these tumours. The next paper, on the diagnosis and clinical manifestations, covers all the possible ways in which this group of tumours may be seen, and lists the procedures which need to be adopted for diagnosis and staging. The place of staging laparotomy is discussed in the next paper, and is summarized well in the final sentence :"Staging laparotomy has a highly selected but infrequent place in the management of the patient with non-Hodgkin's lymphoma." Then there are 2 chapters on the treatment of this lymphoma, summarizing the 2 authors' experience with chemotherapy and radiotherapy. Finally in this section is a description of the condition in children, the way it differs from non-Hodgkin's lymphoma in adults, and the improved survival which has been achieved with intensive chemotherapy.

The second group of papers deals with cranial tumours, mainly in children, and includes the role of radiotherapy in acute lymphoblastic leukaemia. The first of the 3 papers in this section reviews the use of the CT Scanner in diagnosing brain tumours in children. The second paper concerns the radiotherapy and chemotherapy of some brain tumours in children and adults. The final paper reviews the radiation treatment used as prophylaxis for CNS disease in acute lymphoblastic leukaemia, and rightly ends by stressing the need for further improvements in the management of this disease, despite the great improvements already achieved.

The third group of papers relates to genitourinary tumours, picking out some aspects of the management of this group of tumours. The value of radiation therapy in carcinoma of the prostate is assessed and future prospects discussed. The next paper reviews the progress that has been made in the treatment of testicular tumours with chemotherapy, but mention is also made of the use of this modality in other urogenital tumours. The third paper in this group describes the combined radiotherapy and surgical approach to the management of T3 tumours of the bladder.

The fourth group is entitled "Physics in Radiation Oncology" and the first paper usefully describes the use of the computer in radiation therapy. The second paper describes the problem of mycosis fungoides and its clinical features and management, leading appropriately to the last paper about the physical aspects of electron beam therapy in its application to the treatment of mycosis fungoides.

This is an interesting group of papers on a variety of subjects, but seems expensive for a collection of papers many of which have been published elsewhere.

D. Pearson